# Clinical study of the time of repeated computed tomography and replanning for patients with nasopharyngeal carcinoma

**DOI:** 10.18632/oncotarget.16770

**Published:** 2017-03-31

**Authors:** Xiujuan Gai, Yumei Wei, Hengmin Tao, Jian Zhu, Baosheng Li

**Affiliations:** ^1^ School of Medicine and Life Sciences, University of Jinan-Shandong Academy of Medical Sciences, Shandong, China; ^2^ Department of Radiation Oncology VI, Shandong Cancer Hospital Affiliated to Shandong University, Jinon, Shandong, China; ^3^ Shandong Provincial Hospital affiliated to Shandong University, Shandong, China; ^4^ Shandong Provincial Western Hospital, Shandong, China; ^5^ Department of Radiation Oncology, Shandong Cancer Hospital Affiliated to Shandong University, Shandong, China

**Keywords:** nasopharyngeal carcinoma (NPC), cone beam computed tomography (CBCT), gross tumor volume (GTV), parotid gland, displacement

## Abstract

**Purpose:**

To study the necessity of repeat computed tomography (CT) scan and replanning and know a more accurate time using weekly kilovoltage cone beam computed tomography (kV-CBCT) scans for patients with nasopharyngeal carcinoma (NPC) during radiotherapy.

**Methods and Materials:**

Thirteen NPC patients treated with IMRT were enrolled into this prospective study. Weekly pretreatment kV-CBCT scans were performed on the 1^st^, 6^th^, 11^st^, 16^th^, 21^st^ and 26^th^ radiation time, respectively. Target delineations were contoured on all fractionated CBCT images, including the gross tumor volume of the primary nasopharyngeal tumor (GTVnx) and parotid glands. The volumes of GTVnx and parotid glands were calculated automatically using the Pinnacle^3^ 8.0 system. Compared to the original GTVnx, the percentage of shrinking volume (ΔP) ≥ 50% was considered significantly.

**Results:**

As the radiation proceeding, the GTVnx had a trend of shrinkage. Of all 13 patients, 11 cases (84.6%) had the volume shrinking ≥ 50% before the 21^st^ radiation and 12 cases (92.3%) before the 26^th^ radiation. And the parotid volume decreased significantly in the first four-week radiation, 6.45 ± 3.16cm^3^ (range, 3.06-13.9cm^3^) for the left parotid gland and 5.78 ± 2.39cm^3^ (range, 2.70-11.2cm^3^) for the right. Furthermore, only a little displacement occurred to bilateral parotid glands.

**Conclusion:**

The replanning for NPC patients with IMRT is necessary, and the time between the 21^st^ to 25^th^ radiations is appropriate.

## INTRODUCTION

Nasopharyngeal carcinoma (NPC) is commonly seen in endemic populations from some regions of the world, especially, the Southern China. Due to the anatomically challenging location and its high radiosensitivity, radiation therapy (RT) has been recognized as the main curative treatment modality for locoregionally confined NPC [1]. Over the past decade, intensity-modulated radiation therapy (IMRT) has acquired excellent popularity in the treatment of NPC, because of its high efficacy of local control and decreased treatment toxicity. Compared with conventional radiotherapy, IMRT offers improved dose conformity to tumor target coverage with a relative sparing of sensitive normal tissues [2–5]. With the sharp dose drop-offs common to IMRT plans, consistent and accurate dose distribution to tumor targets and their surrounding critical organs are crucial [6, 7]. However, in the course of treatment, most of NPC patients undergoing IMRT have significant changes in anatomical structures, including tumor shrinkage and/or surrounding critical organs shrinkage. And these led to decreased doses to tumor targets and increased to critical structures [8, 9]. For example, the parotid glands undertake the major role in salivary secretion. And xerostomia is one of the most common side effects caused by amount of radiation delivered to parotid glands, which would affect patients’ quality of life. So it is of great significance to grasp changes in volume and displacement of tumor targets and the parotid glands throughout the IMRT treatment course.

With the development of radiotherapy technology, kilovoltage (kV) cone beam computed tomography (CBCT) imaging system installed on a medical linear accelerator has been introduced. It has become an important technique for realizing image-guided radiation therapy. During the course of fractionated radiotherapy, the kV-CBCT could not only assess patient setup errors contrasted with the bony anatomy and soft-tissues, but monitor volume and displacement changes of tumor targets and relative critical organs. A recent study has reported that for NPC patients the second CT scan and replanning before the 25^th^ fraction of IMRT are benefit to ensure appropriate doses to tumor target volumes and surrounding normal tissues [10].

In order to study further the necessity of repeat CT scan and replanning and know the more accurate time for patients with NPC, we aimed at investigating tumor targets’ volume and parotid glands’ volume and displacement changes using weekly kV-CBCT scans during the course of IMRT.

## RESULTS

The volumes of GTVnx on fractionated CBCT images were all shown in Table 2, while Table 3 and Table 4 showed all values of ΔGTV and ΔP, respectively. As the radiation proceeding, the GTVnx had a trend of shrinkage. In the first four-week radiotherapy, the total shrinking volumes (ΔGTV_21_) ranged from 7.2 cm^3^ to 20.7 cm^3^ (mean, 13.7 cm^3^), with the percentage (ΔP_21_) from 19.3% to 69.1% (median, 54.8%). And before the 26^th^ radiation, the shrinking volumes (ΔGTV_26_) ranged from 7.6 cm^3^ to 27.1 cm^3^ (mean, 19.6 cm^3^), with the percentage (ΔP_26_) from 20.4% to 85.3% (median, 82.8%). Meantime, the correlation analysis showed that the percentage of shrinkage was related to the initial volume of GTVnx (r = -0.623, *P* = 0.023), but the absolute decline in values were not connected with it. In addition, of all 13 patients, only one person (1/13) had the volume shrinking ≥ 50% (52%) before the 16^th^ radiation, but 11 cases (84.6%) before the 21^st^ radiation and 12 cases (92.3%) before the 26^th^ radiation, including 11 cases (84.6%) decreased ≥ 70% and 9 cases (69.2%) decreased ≥ 80%. Furthermore, Figure 2 and Figure 3 displayed the tendency of mean ΔGTV and ΔP as the radiation, both with a higher rate between the 21^st^ radiation and the 26^th^ radiation.

**Table 1:** Patient characteristics and tumor stage

Patient characteristic	N
Age (y)	Mean 44 (range, 18–60)
Sex	
Male	9
Female	4
KPS	
100	4
90	5
80	4
T-category	
1	1
2	5
3	4
4a	3
N-category	
0	4
1	6
2	3
Stage group	
III	7
IVa	6

**Table 2:** The volumes of GTVnx of all NPC patients on fractionated CBCT images

V(cm^3^)/No.	GTV1	GTV6	GTV11	GTV16	GTV21	GTV26
1	37.8	30.3	27.5	20.3	17.1	10.9
2	21.5	16.9	14.4	13.1	10.1	3.7
3	20.4	15.5	15.1	9.8	6.3	3.2
4	16.3	12.1	10.6	8.9	7.2	3.8
5	30.4	23.4	21.6	17.8	12.8	4.5
6	22.8	16.8	15.4	12.7	10.3	3.5
7	24.3	18.3	16.5	12.9	11.2	4.1
8	27.2	21.0	17.1	14.3	11.6	4.0
9	18.8	14.1	12.7	11.7	8.6	3.4
10	25.8	18.4	16.5	13.6	11.4	4.8
11	32.4	25.4	21.6	19.8	14.2	5.3
12	37.3	37.1	32.9	32.2	30.1	29.7
13	41.7	35.7	35.4	33.6	27.5	20.4

(GTVnx: Gross tumor volume of primary nasopharyngeal tumor, NPC: Nasopharyngeal carcinoma, CBCT: Cone beam computed tomography, GTV: Gross tumor volume)

**Table 3:** Volumetric changes of GTVnx beween each fractionated volume and the original volume

Δ(cm3)/No	ΔGTV6	ΔGTV11	ΔGTV16	ΔGTV21	ΔGTV26
1	7.5	10.3	17.5	20.7	26.9
2	4.6	7.1	8.4	11.4	17.8
3	4.9	5.3	10.6	14.1	17.2
4	4.2	5.7	7.4	9.1	12.5
5	7.0	8.8	12.6	17.6	25.9
6	6.0	7.4	10.1	12.5	19.3
7	6.0	7.8	11.4	13.1	20.2
8	6.2	10.1	12.9	15.6	23.2
9	4.7	6.1	7.1	10.2	15.4
10	7.4	9.3	12.2	14.4	21.0
11	7.0	10.8	12.6	18.2	27.1
12	0.2	4.4	5.1	7.2	7.6
13	6.0	6.3	8.1	14.2	21.3

(GTVnx: Gross tumor volume of primary nasopharyngeal tumor, ΔGTV: Volumetric changes of GTVnx)

**Table 4:** Percentage of shrinking volume for the fractionated volume compared to the original volume

P(%)/No.	ΔP_6_	ΔP_11_	ΔP_16_	ΔP_21_	ΔP_26_
1	19.8	27.2	46.3	54.8	71.2
2	21.4	33.0	39.1	53.0	82.8
3	24.0	26.0	52.0	69.1	84.3
4	25.8	35.0	45.4	55.8	76.7
5	23.0	28.9	41.4	57.9	85.2
6	26.3	32.5	44.3	54.8	84.6
7	24.7	32.1	46.9	53.9	83.1
8	22.8	37.1	47.4	57.4	85.3
9	25.0	32.4	37.8	54.3	81.9
10	28.7	36.0	47.3	55.8	81.4
11	21.6	33.3	38.9	56.2	83.6
12	0.5	11.8	13.7	19.3	20.4
13	14.4	15.1	19.4	34.1	51.1

(ΔP: Percentage of shrinking volume)

**Figure 1:**
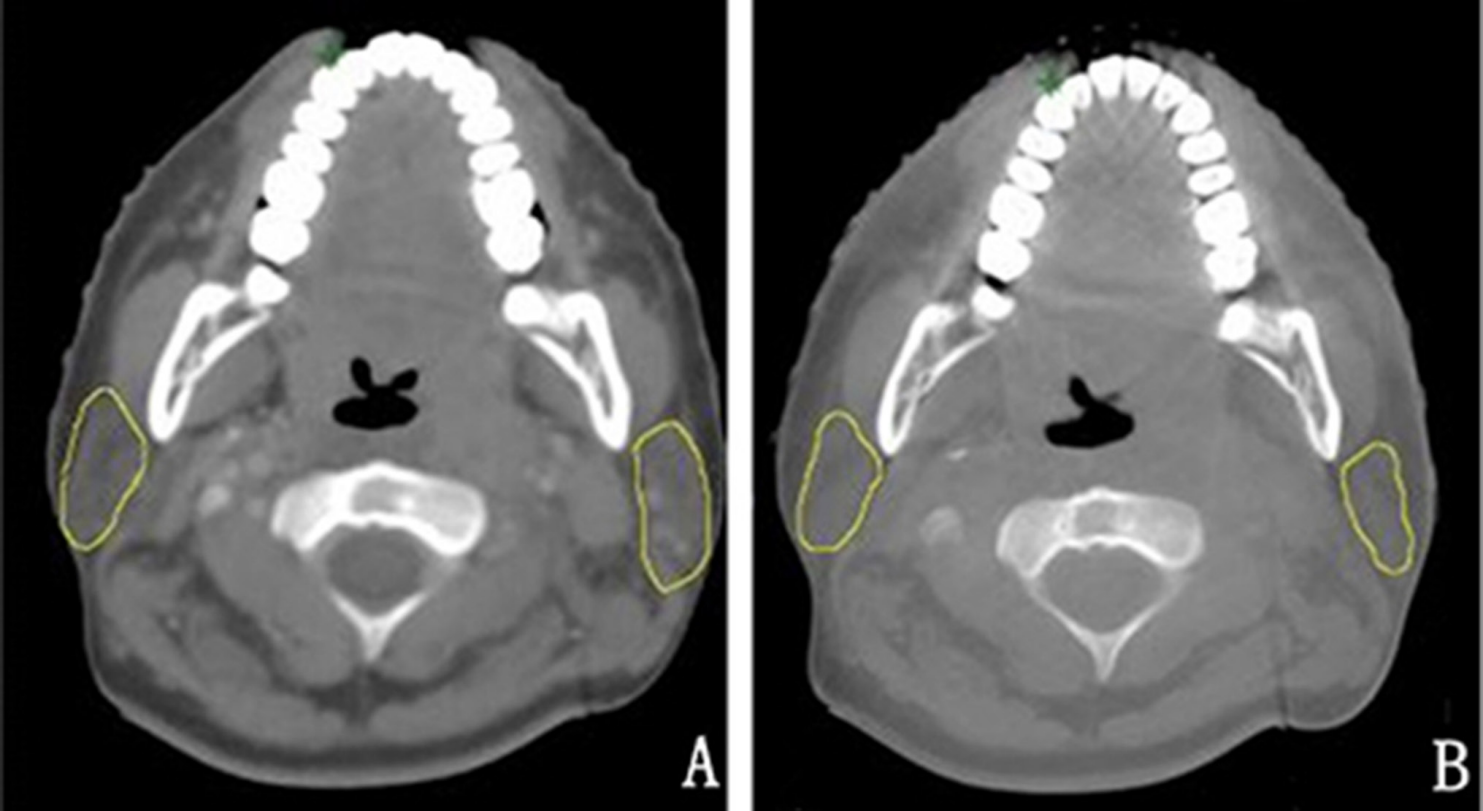
The contrast of the nasopharynx in the transverse section between the planning CT image and CBCT image before the first radiation from one patient **A**. the planning CT **B.** CBCT before the first radiation.

**Figure 2:**
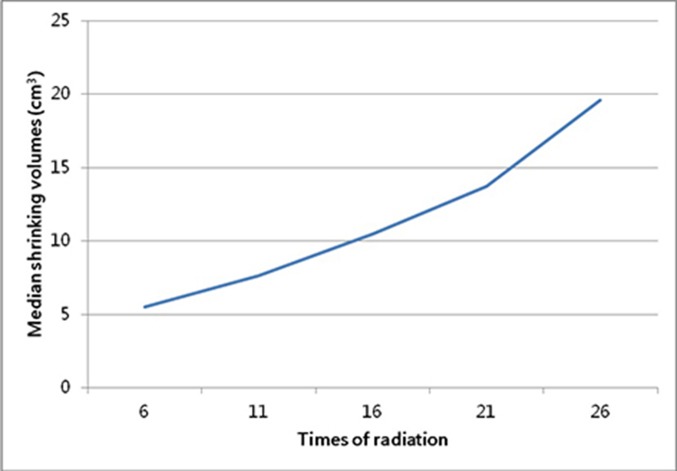
The tendency of median volumetric changes of GTVnx between the fractionated volume and original volume

**Figure 3:**
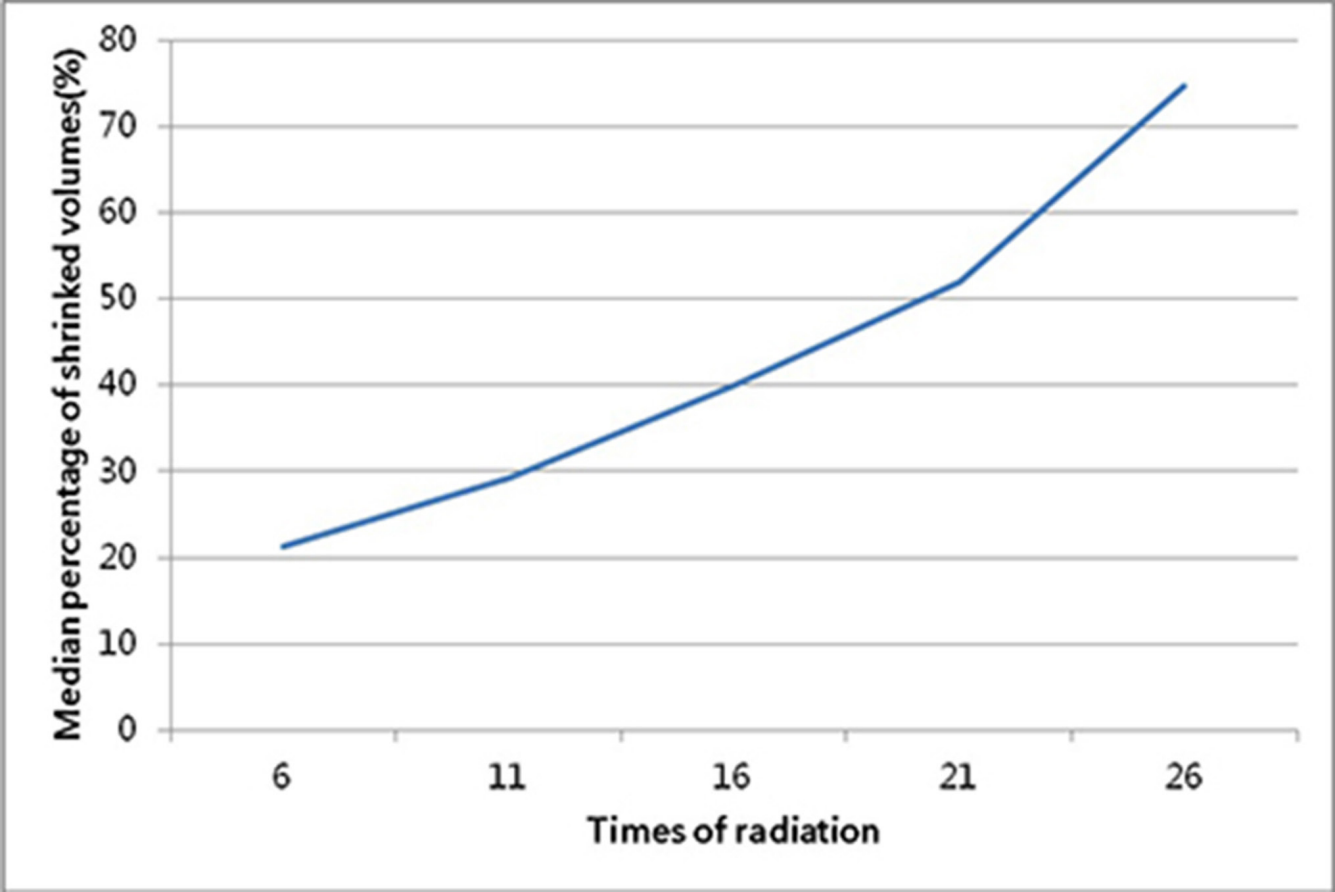
The variation tendency of mean percentage of shrinking volume for the fractionated volume compared to the original volume

**Figure 4:**
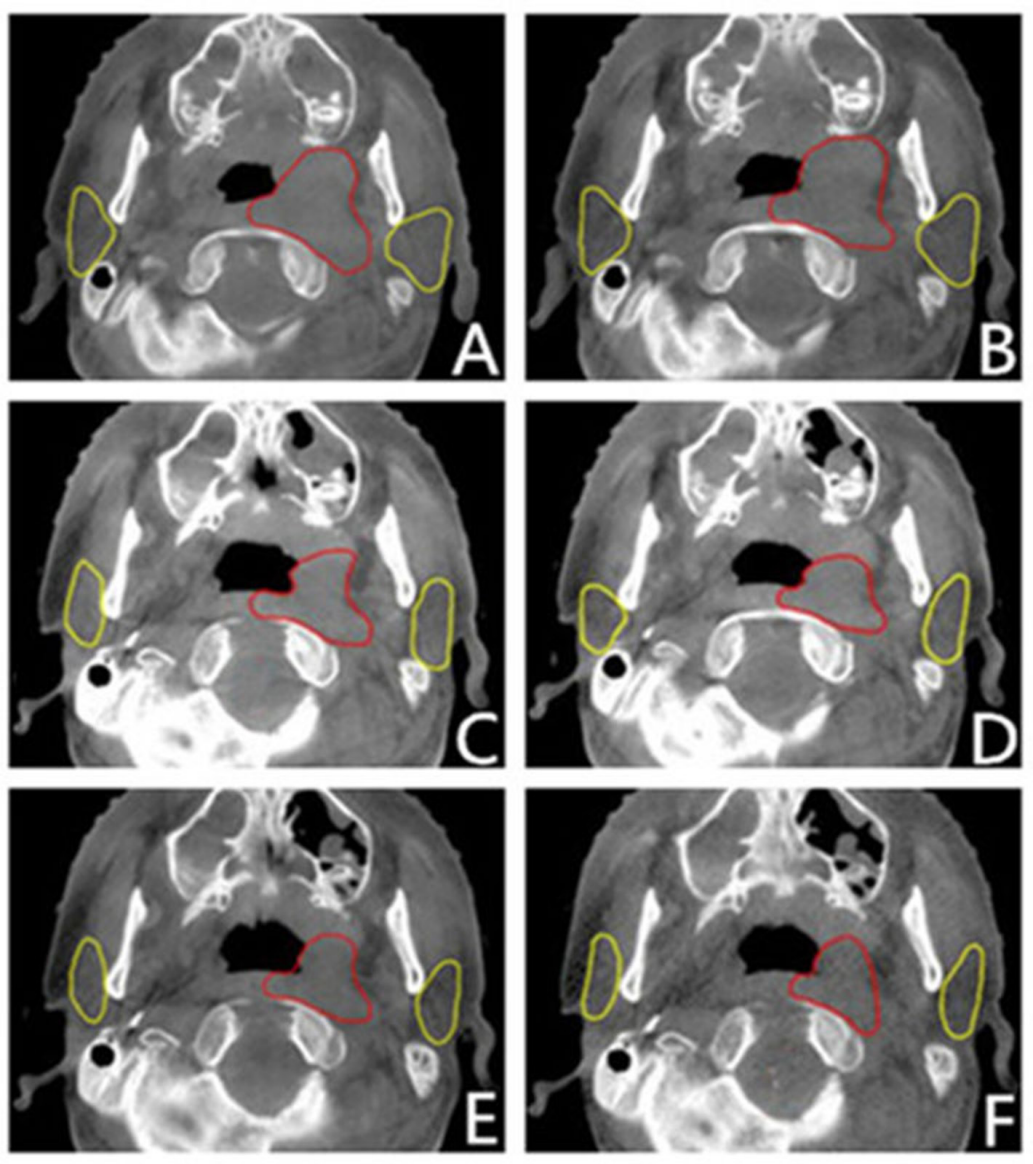
CBCT images performed before the 1 ^st^, 6^th^, 11^st^, 16^th^, 21^st^ and 26^th^ radiation time from one patient, corresponding to letter A, B, C, D, E, F. (In this level, the GTV and parotid glands were showed typically and clearly. The red outlines were GTV, and the yellow were parotids.)

**Figure 5:**
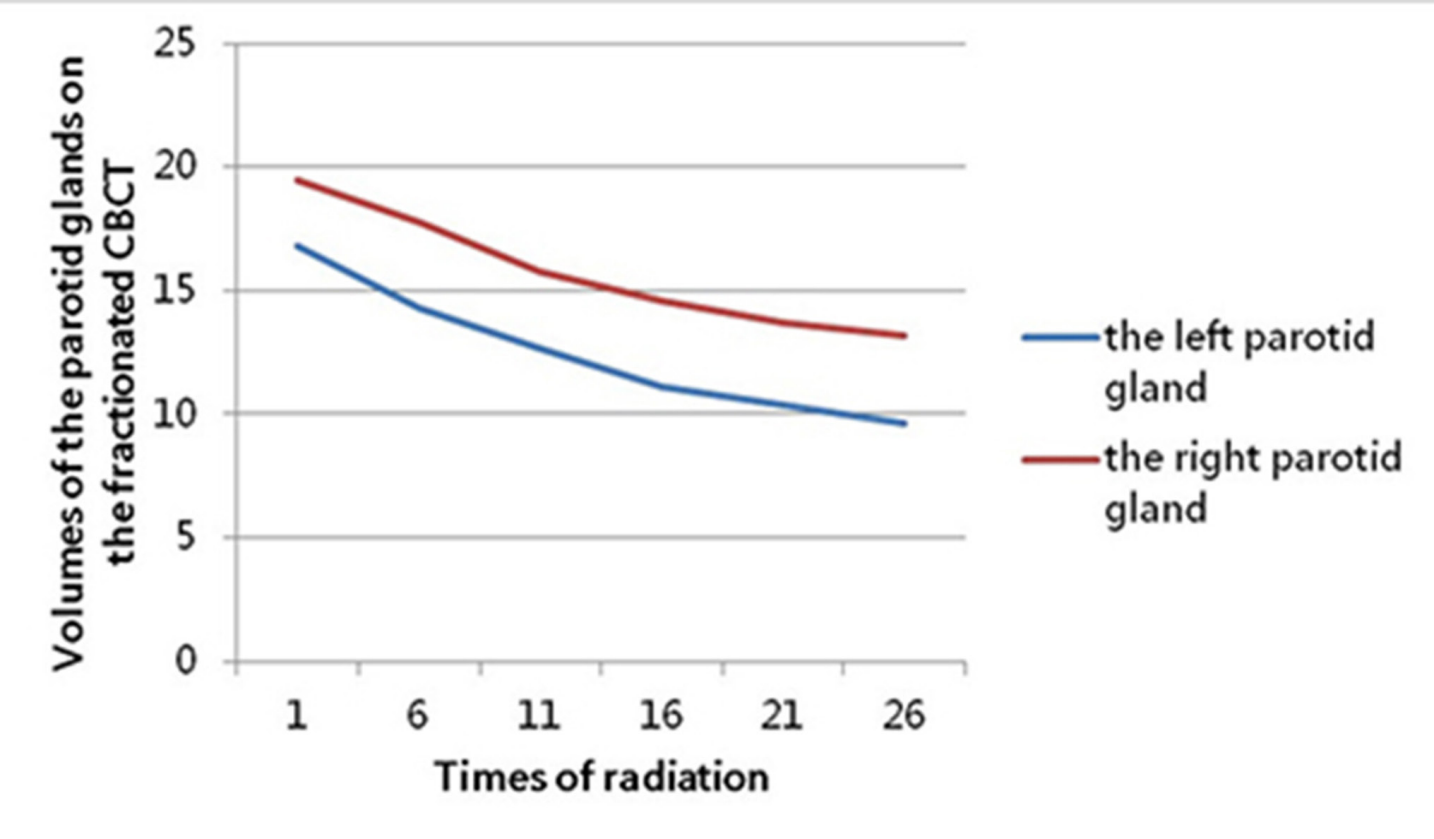
The variation tendency of the bilateral parotid glands’ volume (cm^3^) on the fractionated CBCT

On the other hand, the parotid volume decreased significantly during radiotherapy, as displayed in Figure 4, and with all values showed in Table 5 and Table 6. In the first four-week radiotherapy, the total shrinked volumes (ΔV_21_) of the left and right parotid glands were 6.45 ± 3.16cm^3^ (range, 3.06-13.9cm^3^) and 5.78 ± 2.39cm^3^ (range, 2.70-11.2cm^3^), respectively. And before the 26^th^ radiation, the volume reduction (ΔV_26_) was 7.20 ± 3.33cm^3^ (range, 3.75-14.1cm^3^) and 6.34 ± 2.50cm^3^ (range, 2.88-12.0cm^3^) for the left and right parotid glands, respectively. When measured as a percentage of the initial volume before the 21^st^ radiation, the average volume reduction was 41.7% and 31.6% for the left and right parotid glands, respectively, and before the 26^th^ radiation, the percentage was 46.2% and 34.6%, respectively. Although the decrease in the volume of left parotid is larger than that of right parotid, there were no statistical significance, with *P* = 0.55 and *P* = 0.46 after the first four-week and the 5^th^ week, respectively. No correlation was found between the original volumes and shrinking volumes. But there was obviously positive correlation between the absolute and relative shrinkage of parotid volumes and the planned mean dose (D_mean_) for bilateral parotids, with the value of r and *P* displayed in Table 7. Furthermore, Figure 5 showed the volumetric variation trend of bilateral parotid glands, respectively, with volumes decreasing significantly in the first four-week radiation for bilateral parotid glands.

**Table 5:** Volumes of the left parotid gland of all NPC patients on fractionated CBCT images

V(cm^3^)/No.	1	6	11	16	21	26
1	16.28	14.75	12.91	12.04	11.87	11.20
2	16.86	16.28	15.8	14.54	13.8	13.11
3	26.33	25.22	23.49	23.03	22.67	21.9
4	25.66	24.66	21.99	21.87	21.17	21.04
5	11.23	10.04	8.94	6.46	6.12	5.34
6	16.04	6.49	4.99	2.47	2.10	1.98
7	9.54	7.89	7.80	4.05	3.78	3.18
8	13.42	8.86	5.18	3.15	2.98	2.65
9	22.8	16.78	13.42	13.26	12.52	9.67
10	16.79	15.78	14.65	13.21	10.57	9.34
11	15.43	13.23	12.32	10.45	9.87	9.12
12	13.56	11.76	11.21	10.2	8.65	8.12
13	14.78	13.56	11.89	9.98	8.78	8.45

(NPC: Nasopharyngeal carcinoma, CBCT: Cone beam computed tomography)

**Table 6:** Volumes of the right parotid gland of all NPC patients on fractionated CBCT images

V(cm^3^)/No.	1	6	11	16	21	26
1	17.97	16.9	14.47	13.56	12.53	11.89
2	19.98	17.44	15.89	15.39	14.7	14.1
3	34.12	32.81	29.63	29.48	27.6	26.8
4	24.48	24.42	22.06	21.9	21.78	21.6
5	12.38	10.85	10.37	9.98	8.78	8.56
6	17.92	17.52	13.01	10.13	9.33	9.04
7	11.59	11.28	10.32	9.34	8.76	8.10
8	15.88	9.51	6.82	5.93	4.68	3.84
9	20.34	16.51	14.57	13.84	13.52	13.42
10	18.9	16.78	15.46	13.78	11.29	10.34
11	25.34	23.98	21.76	20.67	19.98	19.21
12	12.67	11.89	10.45	8.78	8.21	7.89
13	21.67	20.56	19.67	17.21	16.89	16.01

(NPC: Nasopharyngeal carcinoma, CBCT: Cone beam computed tomography)

**Table 7:** The correlation between the shrinkage of parotid volume and the planned mean dose for bilateral parotids

Shrinkage	Times of radiation	The left parotid		The right parotid	
		r	*P*	r	*P*
**Absolute**	21^th^	0.934	0.001*	0.916	0.001*
	26^th^	0.842	0.009*	0.925	0.001*
**Relative**	21^th^	0.812	0.014	0.845	0.008*
	26^th^	0.769	0.018	0.850	0.008*

(Absolute: the shrinking volumes of parotids, Relative: the percentage of shrinking volumes compared with the original volumes, r: Pearson correlation coefficient, ”*”: Two-sided P-values of < 0.01 were considered statistically significant.)

**Figure 6:**
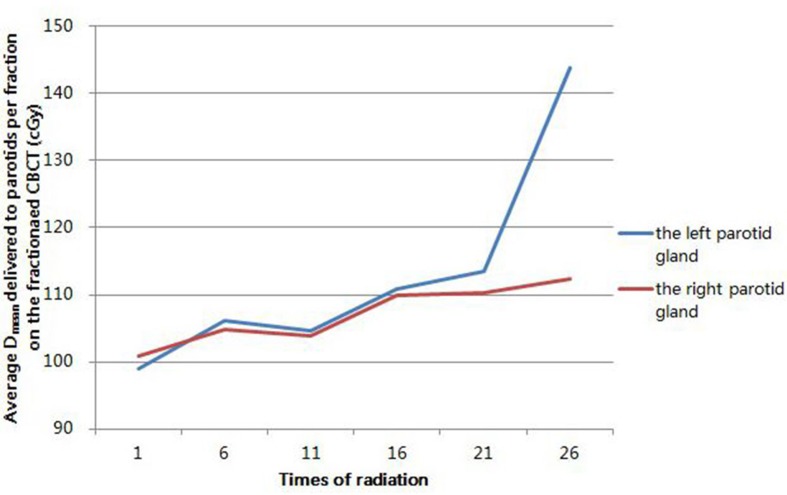
The variation trend of average mean doses (D^mean^) delivered to bilateral parotid glands per fraction on the fractionated CBCT

Figure 6 displayed the variation trend of average D_mean_ delivered to bilateral parotid glands per fraction, respectively. It showed the tendency of rising after the 11^st^ radiation. And the percentages of increased average D_mean_ of the left parotid gland were 14.6% and 45.3% at the 21^st^ and 26^th^ radiation, respectively, with 9.4% and 11.4% for the right. Meanwhile, median displacement of the parotid glands were displayed in Table 8. Only a little displacement occurred to bilateral parotid glands. But a trend to shift medially for both the left and right parotid gland could also been demonstrated.

**Table 8:** Median distances between the parotid COM and the atlas COM

Times of treatment	Distances between the parotid COM and the atlas COM (cm)	
	Left Parotid	Right Parotid
1	7.14	7.29
6	7.12	7.24
11	7.11	7.23
16	7.03	7.21
21	7.01	7.20
26	6.93	7.16

(COM: Center of mass)

## DISCUSSION

Over the past decade, IMRT has become the routine treatment for patients with NPC disease. But most NPC patients would experience anatomic changes during the course of IMRT, mainly including shrinkage of the primary tumor and displacement changes of parotid glands [8–10]. Due to the sharp dose gradient of IMRT technique, these changes could cause a potential dosimetric impact, including decreased doses to tumor targets and increased to normal structures. Therefore, repeat CT scan and replanning are necessary. A previous study has reported that repeat CT scan and replanning before the 25^th^ radiation would significantly increase the tumor target dose coverage and reduce the normal structures’ doses [10]. However, all repeat CT scan and replanning in this study were performed only before the 25^th^ radiation, compared to another study with an average interval of 39 ± 11 days (average, 19 ± 6 fractions) between the first radiation and the repeat CT scan [11]. To monitor changes of the tumor and critical organs timely during the process of radiotherapy and know a more accurate time for repeat CT scan and replanning, we conducted a pilot study by using the kV-CBCT weekly. And in our study, the shrinking volumes and percentage of GTVnx in the first four-week radiotherapy were similar to the result of another previous study, with the range from 5.1cm^3^ to 21.4cm^3^ (median, 9.85cm^3^) and from 23.0% to 58.2% (median, 27.15%), respectively [12]. We also demonstrated that a significant shrinkage ( ≥ 50%) occurred after the 20^th^ radiation, and a substantial proportion of reduction then happened between the 21^st^ and 25^th^ radiation. As a result, replanning between the 21^st^ and 25^th^ fraction during the IMRT is recommended.

Not only could changes in organ anatomy and tumor size cause target underdose during IMRT, but also overdose to OARs could lead to additional complications, like xerostomia caused by high radiation dose of exposure to parotid glands. Although the incidence of xerostomia reduced with the use of IMRT technique, volumetric reduction and displacement change could also result in severe dry mouth symptoms, affecting the quality of life for patients with NPC disease [13, 14]. In our study, the parotid volume decreased gradually during radiotherapy, with the average reduction of 41.7% and 31.6% for the left and right parotid glands in the first four-week and of 46.2% and 34.6% after the 5^th^ week. However, another recent study reported that the average volume reduction was 29.47% and 24.47% for the left and right parotid glands at the end of radiation therapy [15], which were smaller than our results. And previously published studies demonstrated that parotid volumes had decreased little during the first 3-4 weeks [16, 17] and stabilized after the 25^th^ radiation [14]. Maybe the use of helical tomotherapy and radiotherapy combined with different chemotherapy or anti-EGFR Mab could explain it.

We found a rising trend of the average D_mean_ for bilateral parotids per fraction, with increased percentages of 45.3% and 11.4% for the left and right parotid glands at the 26^th^ radiation, respectively. And a previous study reported a similar result, with the accumulative average D_mean_ increasing of 11.38% at the end of radiation therapy for all enrolled parotid glands with helical tomotherapy [18]. The sharp increase of the average D_mean_ per fraction for the left parotid gland could be caused by the location of the primary tumors, which were mostly (8/13) located on the left side of the body. In the light of the previous study, replanning was regarded to be necessary if absolute parotid doses were more than 10% compared to the initial planning dose. In our research, according to the shrinkage of GTVnx and the D_mean_ raise of parotids, it is necessary to execute replanning between the 21^st^ and 25^th^ fraction during the IMRT.

Similar to previous studies [19, 20], displacement changes in the parotid glands were not obvious in our study, which aimed at the displacement of parotid centers. Robar et al. [19] has reported that the parotid centers remained unchanged, though the outside boundaries shifted to the midline (with an average of 2.6 mm for the left parotid gland and 1.9 mm for the right parotid gland). And Vasquez Osorio et al [20] also found that displacement changes in the central region of parotid glands were minimal. We think that the changes in the peripheral region could be explained by the volumetric reduction of bilateral parotid glands. The traction caused by the shrinkage of tumors may result in the displacement of parotid centers. Most tumors (8/13) in our research were located on the left side of the body. And this could make the traction for the left parotid glands more than the one for the right, with the displacement of 0.21cm for the left parotid and 0.13cm for right after the 26^th^ radiation. But we think there was no significance for the little difference (0.08cm).

## MATERIALS AND METHODS

### Patient characteristics

From January 2014 to April 2015, a total of 13 newly diagnosed patients at the Shandong Cancer Hospital Affiliated to Shandong University were enrolled into this prospective study. Eligible patients were individuals with histologically proven and locoregionally advanced (stage III-IVa) NPC. The disease was staged according to the 7th American Joint Commission on Cancer staging system. Patients specific characteristics are summarized in Table 1. CBCT is a generally accepted technique to monitor variations and decrease setup errors for NPC patients undergoing IMRT, so the authors advise that the Institutional Review Board deemed the study violated no ethics and the ethics approval was not imperative. Informed consent was provided by all patients before participation.

### Radiotherapy simulation

Patients were immobilized with a thermoplastic head-and-shoulder mask in the supine position. Imagings were scanned with a slice thicknesses of 3 mm on a computed tomography simulator. Each scan was performed from the vertex to below the clavicles. The CT datasets were transmitted to the Pinnacle^3^ 8.0 workstation through DICOM network for contouring targets and organs at risk (OARs).

### Target delineation and treatment planning

The target delineation was contoured on the simulation CT images by one oncologist and confirmed by another oncologist. The gross tumor volume (GTV) included the primary nasopharyngeal tumor (GTVnx) and involved lymph nodes (GTVnd) as shown in the enhanced CT images, magnetic resonance imaging images and/or positron emission tomography. The clinical target volume 1 (CTV1) was defined as high-risk regions surrounding the primary tumor (including the entire nasopharynx, skull base, parapharyngeal space, posterior third of the nasal cavity and maxillary sinuses, pterygopalatine fossa, inferior sphenoid sinus, posterior ethmoid sinus and anterior half of the clivus) and all high-risk neck nodes. CTV2 was defined as low-risk node regions below the CTV1. The respective planning target volumes (PTVs) were generated with a margin of 5mm in all directions, but with a 2-3mm margin when the corresponding CTV has overlapped with, or been close to, a critical structure, such as brain stem, spinal cord or optic nerves.

The prescribed radiation dose was delivered in 33 fractions, with 2.2Gy per fraction to PTVnx and PTVnd, 2.0Gy to PTV1 and 1.8Gy to PTV2. All patients were treated with one fraction daily for five days weekly. Radiation was delivered using a 6-MV linear accelerator (Varian Medical Systems, Palo Alto, CA, USA) once the treatment planning was approved. Furthermore, all patients were typically treated with cisplatin-based concurrent chemotherapy for sensibilization, infused intravenously at 75mg/m^2^, d1-3, two cycles totally.

### CBCT imaging and CBCT guidance protocol

The kV-CBCT images were acquired using the Varian Medical Systems linear accelerator equipped with kV imaging capabilities (Varian Medical Systems, Palo Alto, CA, USA), with the following acquisition parameters: kVp, 100 kV; nominal milliamperes per frame, 10 mA; nominal milliseconds, 10 ms; kV collimator, s20; kV filter, f0; approximate frames, 361; and total angle, 200. Figure 1 shows the contrast between CBCT images and planning CT images of the nasopharynx in the transverse section. It is obvious that the CBCT images have the quality needed to perform an accurate image registration with planning CT images.

The kV-CBCT scans were performed before the 1^st^, 6^th^, 11^st^, 16^th^, 21^st^ and 26^th^ radiation time, respectively, after conventional positioning by calibrating the in-room lasers on the marked thermoplastic mask.

### Target delineation on CBCT images

All fractionated CBCT images before the treatment were delivered to the Pinnacle^3^ 8.0 workstation, fused with the planning CT using the adaptive software. Target delineations (including the GTVnx, bilateral parotids and atlas) were contoured and confirmed by the same oncologist. Then the center of mass (COM) of the atlas and bilateral parotids, volumes of GTVnx and parotid glands, doses delivered to parotids were calculated automatically. The COM was a fictitious point in the physical system where quality was considered to set.

### Statistical analysis

All target volumes delineated on fractionated CBCT images were recorded. Compared with the original GTVnx, it was considered as significant if the percentage of shrinking volume (ΔP) ≥ 50% (ΔP = ΔGTV/ the volume of the original GTVnx, ΔGTV = the volume of GTVnx on the corresponding fractionated CBCT images - the volume of the original GTVnx). Shrinking volumes and the percentage of bilateral parotids were calculated by the same means of the GTVnx. In addition, the space coordinates of all COM points were calculated automatically using the Pinnacle^3^ 8.0 system. The atlas reference point was defined as the coordinate origin, with the space coordinate named point P_1_ (x_1_, y_1_, z_1_). And the distances between each parotid COM [P_2_ (x_2_, y_2_, z_2_)] and atlas COM were cacalculated using the formula [(x_2_-x_1_)^2^+(y_2_-y_1_)^2^+ (z_2_-z_1_)^2^]^1/2^. Then, the displacement was achieved comparing difference between two distances. x2-x12+y2-y12+ z2-z12

The data were analyzed with SPSS version 17.0 statistical software (SPSS Inc., Chicago, IL, USA). The Pearson correlation coefficient was used to evaluate the correlation. Two-sided *P*-values of < 0.05 were considered statistically significant.

## CONCLUSION

In summary, during the IMRT of NPC, volumes of tumor targets and parotid glands varied significantly, with little displacement change in the center of parotid glands. This could cause a decrease radiation dose delivered to tumor targets and an increase radiation dose to parotid glands. So it is necessary to identify the time of repeat CT and replanning, and the time between the 21^st^ to 25^th^ radiations of IMRT is appropriate.

## Abbreviations

NPC: nasopharyngeal carcinoma
RT: radiation therapy
IMRT: intensity-modulated radiation therapy
kV: kilovoltage
CBCT: cone beam computed tomography
GTV: gross tumor volume
GTVnx: GTV included the primary nasopharyngeal tumor
GTVnd: GTV included the involved lymph nodes
CTV: clinical target volume
PTV: planning target volume
D_mean_: the planned mean dose
OARs: organs at risk
COM: the center of mass
ΔP: the percentage of the shrinking volumes
ΔV: the shrinking volumes of parotid glands
ΔGTV: the shrinking volumes of GTVnx
